# Supplementary material for: Role of grain-level chemo-mechanics in composite cathode degradation of solid-state lithium batteries
Source: Nat Commun. 2024 Sep 12;15:7970. doi: 10.1038/s41467-024-52123-w (PMC11393410; doi:10.1038/s41467-024-52123-w)
Supplement: Supplementary file 1 — Supplementary Information [file 41467_2024_52123_MOESM1_ESM.pdf]

Supplementary: Role of grain-level chemo-mechanics in composite cathode degradation of solid-state lithium batteries

Chuanlai Liu*, Franz Roters and Dierk Raabe*

Max-Planck-Institut für Eisenforschung GmbH, Max-Planck-Str. 1, Düsseldorf, 40237, Germany.

*Corresponding author(s). E-mail(s): c.liu@mpie.de; d.raabe@mpie.de;

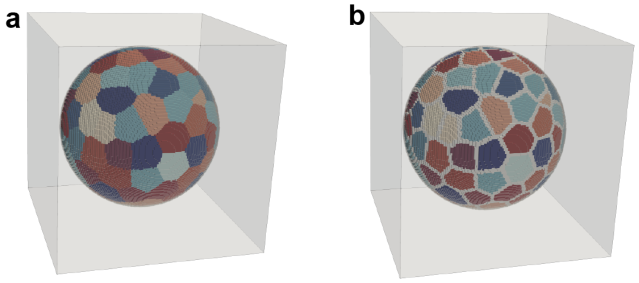

Fig. S.1 Representative volume element describing the microstructure of composite cathodes, without (a) and with (b) grain boundaries.

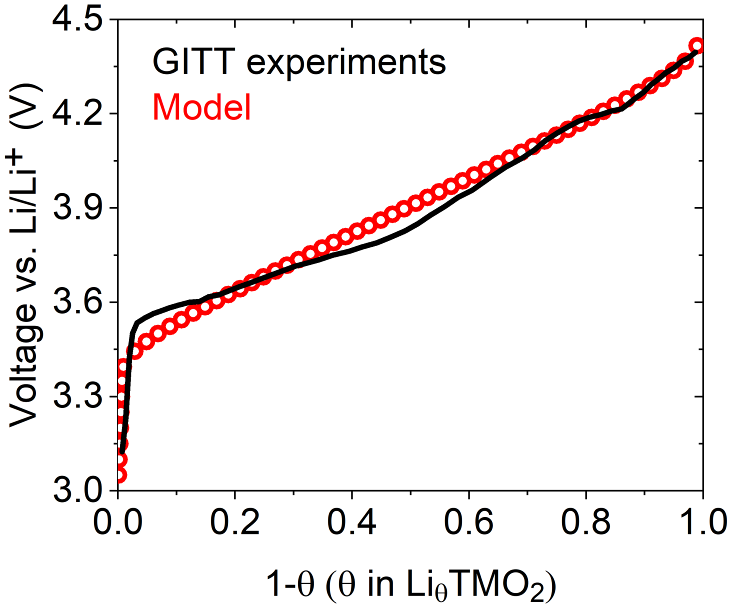

Fig. S.2 Open circuit voltage. The black line shows the results measured at different states of charge from the galvanostatic intermittent titration technique experiment [1]. A regular solution model (red data points) is used to fit this data for use in the chemo-mechanical simulations.

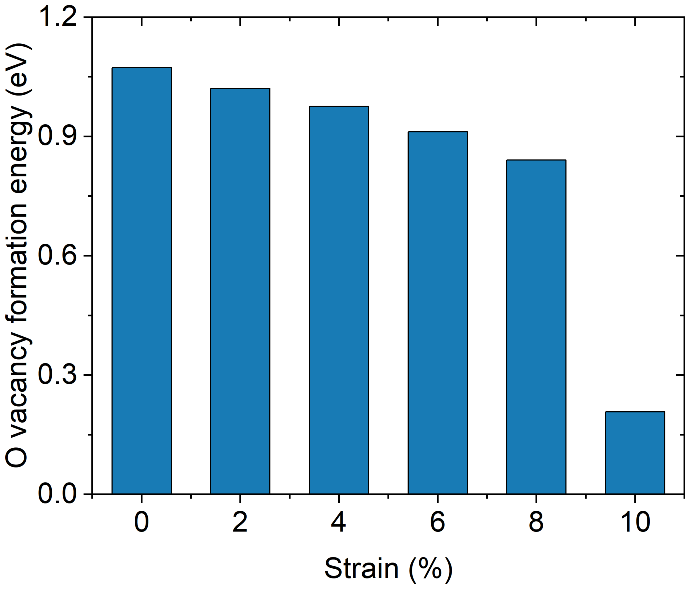

Fig. S.3 Effect of applied tensile strain on the formation energy of oxygen vacancies in the layered oxide cathode [2].

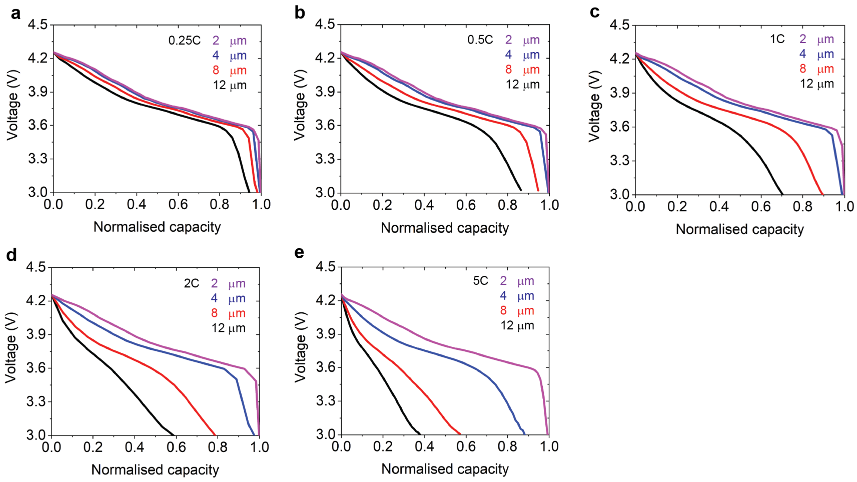

Fig. S.4 Effect of the NMC811 secondary particle size on voltage curves under various C-rates. The modeling C-rate is 0.25C (a), 0.5C (b), 1C (c), 2C (d), and 5C (e), respectively.

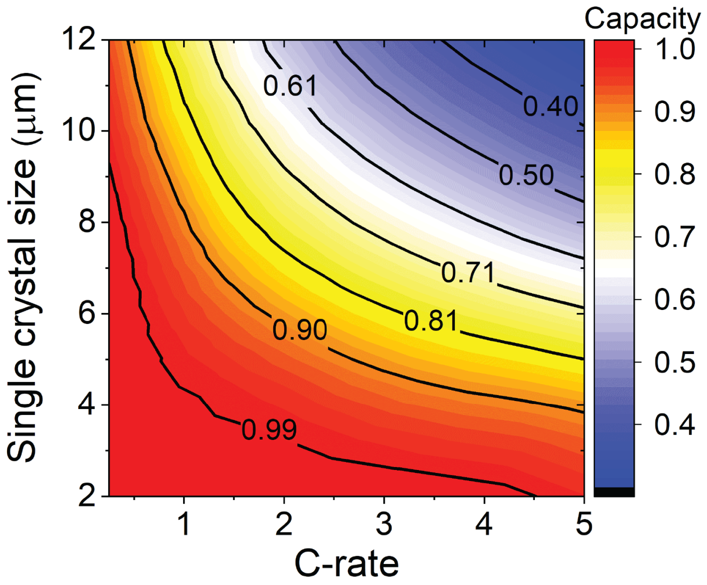

Fig. S.5 Effect of the particle size and C-rate on the normalised capacity of NMC single crystal cathodes.

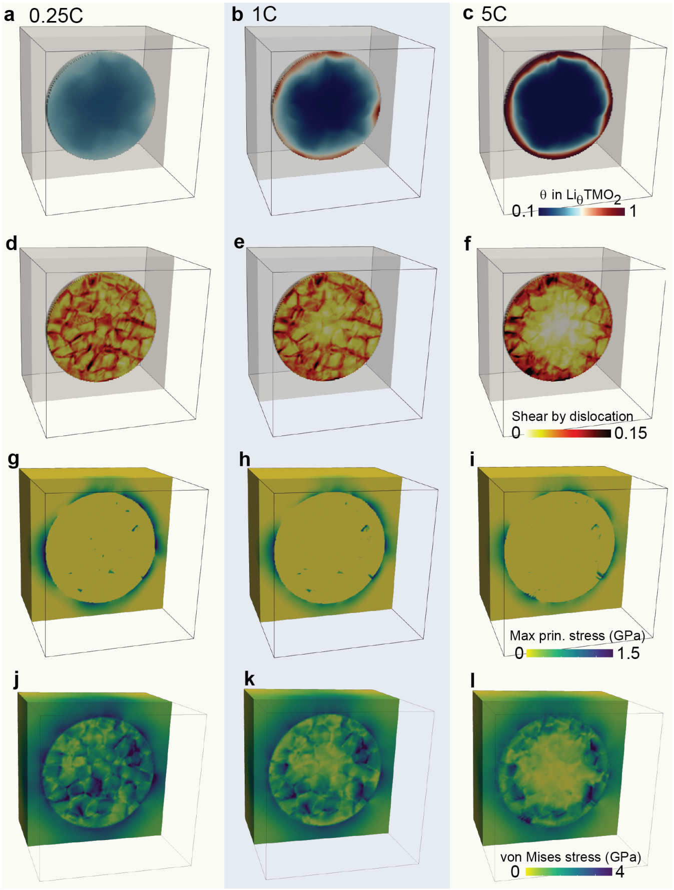

Fig. S.6 Effect of the C-rate on the distribution of Li concentration (a-c), basal dislocation-induced plastic shear (d-f), maximum principle stress (g-i), and von Mises stress (j-l) within the polycrystal cathode particle. The average Li site concentration in the cathode is 40%. The diameter of the NMC811 secondary particle is 12 μm.

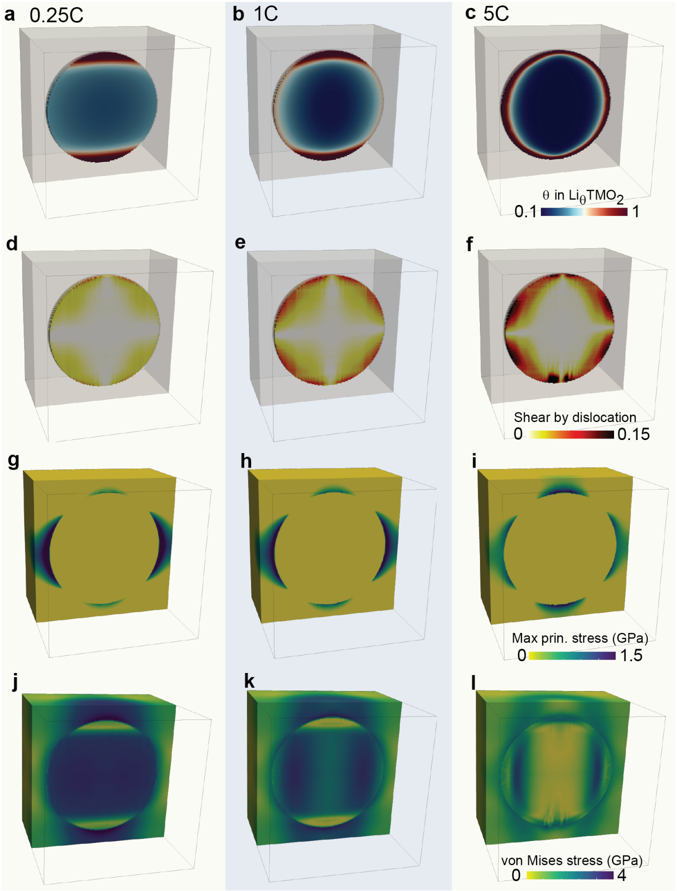

Fig. S.7 Effect of the C-rate on the distribution of Li concentration (a-c), basal dislocation-induced plastic shear (d-f), maximum principle stress (g-i), and von Mises stress (j-l) within the single crystal cathode particle. The average Li site concentration in the cathode is 40%. The diameter of the NMC811 single crystal particle is 12 μm.

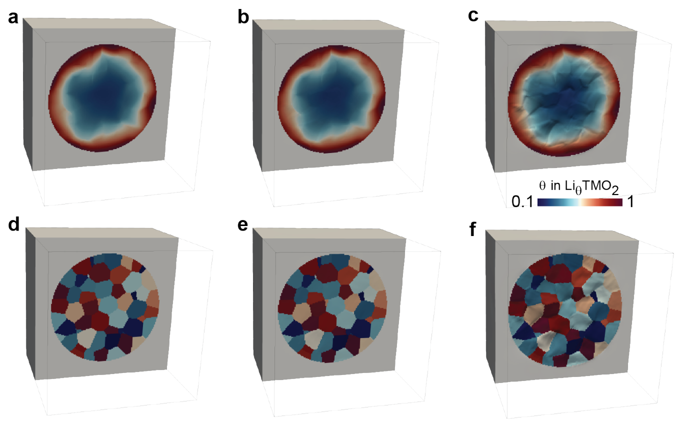

Fig. S.8 Distribution of Li concentration (a-c) and grain morphology (d-f) within the polycrystal secondary particle: reference configuration (a, d), deformed configuration (b, e), and deformed configuration with displacements scaled by a factor of ten (c, f). The average Li site concentration in the cathode is 60%, discharged at 1 C. The diameter of the particle is 12 μm.

Table S1 Material parameters for the chemo-mechanical models.

Chemical free energy [1, 3]	$k_B(\mathrm{Jmol^{-1}K^{-1}})$	T (K)	$F(\mathrm{sAmol^{-1}})$	$E_1(\mathrm{Jmol^{-1}})$	$E_{11}(\mathrm{Jmol^{-1}})$
	8.314	298	96485.3	-4.15×10^5	3.8×10^4
	$C_{\max}(\mathrm{mol\ m^{-3}})$				
	49200				
Diffusivity [1, 3]	$D_{\mathrm{ref}}(\mathrm{m^{-2}s^{-1}})$	D_0	D_1	D_2	D_3
	1×10^{-8}	-17.94	202.3	-782.6	1483
	D_4	D_5			
	-1362	473.6			
Lattice dimension change [1, 4, 5]	$\nu_0^{\mathrm{a,b}}$	$\nu_1^{\mathrm{a,b}}$	$\nu_2^{\mathrm{a,b}}$	$\nu_3^{\mathrm{a,b}}$	$\nu_4^{\mathrm{a,b}}$
	-6.56×10^{-6}	3.143×10^{-3}	-4.885×10^{-2}	0.2774	-0.3405
	$\nu_5^{\mathrm{a,\ b}}$				
	0.1302				
	ν_0^{c}	ν_1^{c}	ν_2^{c}	ν_3^{c}	ν_4^{c}
	-5.157×10^{-2}	0.6483	-1.569	1.512	-0.5208
Crystal plasticity model	$\dot{\gamma}_0(\mathrm{s^{-1}})$	n	g_0 (MPa)	g_∞ (MPa)	a
Basal dislocation [6]	1×10^{-3}	20	39	86	2.25
	h_0 (MPa)	$h_{\alpha\beta}$ (MPa)			
	75	1			
Elastic constants					
NMC811 cathode (lithiated) [7–9]	C_{11}(Pa)	C_{33}(Pa)	C_{44}(Pa)	C_{12}(Pa)	$C_{13}(Pa)$
	1.95×10^{11}	1.77×10^{11}	7.41×10^{10}	1.34×10^{11}	1.19×10^{10}
NMC811 cathode (delithiated) [8, 9]	C_{11}(Pa)	C_{33}(Pa)	C_{44}(Pa)	C_{12}(Pa)	$C_{13}(Pa)$
	2.8×10^{11}	1.8×10^{10}	1.5×10^{10}	7.5×10^{10}	0.8×10^{10}
Oxide electrolyte [10, 11]	C_{11}(Pa)	C_{12}(Pa)	C_{44}(Pa)		
	1.97×10^{11}	8.03×10^{10}	5.81×10^{10}		
Sulfide electrolyte [12]	C_{11}(Pa)	C_{12}(Pa)	C_{44}(Pa)		
	3.87×10^{10}	1.58×10^{10}	1.14×10^{10}		
Polymer electrolyte [13]	C_{11}(Pa)	C_{12}(Pa)	C_{44}(Pa)		
	1.31×10^{9}	5.35×10^{8}	3.88×10^{8}		

Supplementary References

[1] Marker, K., Reeves, P.J., Xu, C., Griffith, K.J., Grey, C.P.: Evolution of structure and lithium dynamics in $LiNi_{0.8}Mn_{0.1}Co_{0.1}O_2$ (NMC811) cathodes during electrochemical cycling. Chemistry of Materials **31**(7), 2545–2554 (2019)

[2] Liu, T., Liu, J., Li, L., Yu, L., Diao, J., Zhou, T., Li, S., Dai, A., Zhao, W., Xu, S., *et al.*: Origin of structural degradation in Li-rich layered oxide cathode. Nature **606**(7913), 305–312 (2022)

[3] Xu, C., Merryweather, A.J., Pandurangi, S.S., Lun, Z., Hall, D.S., Deshpande, V.S., Fleck, N.A., Schnedermann, C., Rao, A., Grey, C.P.: Operando visualization of kinetically induced lithium heterogeneities in single-particle layered Ni-rich cathodes. Joule **6**(11), 2535–2546 (2022)

[4] Xu, C., Märker, K., Lee, J., Mahadevegowda, A., Reeves, P.J., Day, S.J., Groh, M.F., Emge, S.P., Ducati, C., Layla Mehdi, B., *et al.*: Bulk fatigue induced by surface reconstruction in layered Ni-rich cathodes for Li-ion batteries. Nature Materials **20**(1), 84–92 (2021)

[5] Xu, C., Reeves, P.J., Jacquet, Q., Grey, C.P.: Phase behavior during electrochemical cycling of Ni-rich cathode materials for Li-ion batteries. Advanced Energy Materials **11**(7), 2003404 (2021)

[6] Stallard, J.C., Vema, S., Hall, D.S., Dennis, A.R., Penrod, M.E., Grey, C.P., Deshpande, V.S., Fleck, N.A.: Effect of lithiation upon the shear strength of NMC811 single crystals. Journal of The Electrochemical Society **169**(4), 040511 (2022)

[7] Sharma, N., Meng, D., Wu, X., Vasconcelos, L.S., Li, L., Zhao, K.: Nanoindentation measurements of anisotropic mechanical properties of single crystalline NMC cathodes for Li-ion batteries. Extreme Mechanics Letters **58**, 101920 (2023)

[8] Xu, R., Sun, H., Vasconcelos, L.S., Zhao, K.: Mechanical and structural degradation of $LiNi_xMn_yCo_zO_2$ cathode in Li-ion batteries: an experimental study. Journal of The Electrochemical Society **164**(13), 3333 (2017)

[9] Lim, J.-M., Kim, H., Cho, K., Cho, M.: Fundamental mechanisms of fracture and its suppression in Ni-rich layered cathodes: Mechanics-based multiscale approaches. Extreme Mechanics Letters **22**, 98–105 (2018)

[10] Kim, Y., Jo, H., Allen, J.L., Choe, H., Wolfenstine, J., Sakamoto, J.: The effect of relative density on the mechanical properties of hot-pressed cubic Li_7 La_3Zr_2 O_{12}. Journal of the American Ceramic Society **99**(4), 1367–1374 (2016)

[11] Cho, Y.-H., Wolfenstine, J., Rangasamy, E., Kim, H., Choe, H., Sakamoto, J.: Mechanical properties of the solid Li-ion conducting electrolyte: $Li_{0.33}La_{0.57}TiO_3$.

Journal of Materials Science **47**, 5970–5977 (2012)

[12] Kato, A., Nose, M., Yamamoto, M., Sakuda, A., Hayashi, A., Tatsumisago, M.: Mechanical properties of sulfide glasses in all-solid-state batteries. Journal of the Ceramic Society of Japan **126**(9), 719–727 (2018)

[13] Ramesh, S., Winie, T., Arof, A.: Investigation of mechanical properties of polyvinyl chloride–polyethylene oxide (PVC–PEO) based polymer electrolytes for lithium polymer cells. European Polymer Journal **43**(5), 1963–1968 (2007)
